# Continuous-Flow Separation of Magnetic Particles from Biofluids: How Does the Microdevice Geometry Determine the Separation Performance?

**DOI:** 10.3390/s20113030

**Published:** 2020-05-27

**Authors:** Cristina González Fernández, Jenifer Gómez Pastora, Arantza Basauri, Marcos Fallanza, Eugenio Bringas, Jeffrey J. Chalmers, Inmaculada Ortiz

**Affiliations:** 1 Department of Chemical and Biomolecular Engineering, ETSIIT, University of Cantabria, Avda. Los Castros s/n, 39005 Santander, Spain; gonzalezferc@unican.es (C.G.F.); arantza.basauri@unican.es (A.B.); marcos.fallanza@unican.es (M.F.); eugenio.bringas@unican.es (E.B.); 2 William G. Lowrie Department of Chemical and Biomolecular Engineering, The Ohio State University, 151 W. Woodruff Ave., Columbus, OH 43210, USA; gomezpastora.1@osu.edu (J.G.P.); chalmers.1@osu.edu (J.J.C.)

**Keywords:** particle magnetophoresis, CFD, cross section, chip fabrication

## Abstract

The use of functionalized magnetic particles for the detection or separation of multiple chemicals and biomolecules from biofluids continues to attract significant attention. After their incubation with the targeted substances, the beads can be magnetically recovered to perform analysis or diagnostic tests. Particle recovery with permanent magnets in continuous-flow microdevices has gathered great attention in the last decade due to the multiple advantages of microfluidics. As such, great efforts have been made to determine the magnetic and fluidic conditions for achieving complete particle capture; however, less attention has been paid to the effect of the channel geometry on the system performance, although it is key for designing systems that simultaneously provide high particle recovery and flow rates. Herein, we address the optimization of Y-Y-shaped microchannels, where magnetic beads are separated from blood and collected into a buffer stream by applying an external magnetic field. The influence of several geometrical features (namely cross section shape, thickness, length, and volume) on both bead recovery and system throughput is studied. For that purpose, we employ an experimentally validated Computational Fluid Dynamics (CFD) numerical model that considers the dominant forces acting on the beads during separation. Our results indicate that rectangular, long devices display the best performance as they deliver high particle recovery and high throughput. Thus, this methodology could be applied to the rational design of lab-on-a-chip devices for any magnetically driven purification, enrichment or isolation.

## 1. Introduction

Applications of superparamagnetic, micron-sized particles, often called magnetic beads (MBs), have proliferated in the last two decades, most notably in fields related to biomedical science and technology. These materials pose multiple advantages, including but not limited to, high surface-area-to-volume ratio, biocompatibility and chemical stability after treating their surface with coatings, and most importantly, superparamagnetism, which allows their separation from the solution by using simple permanent magnets [1,2,3,4]. As a result, these materials have been successfully applied in multiple processes within the chemical, biomolecular and medical fields [5,6]. For this purpose, the beads are properly synthesized with surface ligands that attach to a multitude of non-magnetic chemical and biological entities [2,3,7,8]. In most of the applications, particularly within sensing and diagnosis, the beads are designed to bind to a target analyte present in low concentration in a highly heterogeneous biological matrix. Due to the low concentration of such analytes, the existing interferences in complex media (i.e., blood or saliva), the detection sensitivity and the instrumental detection limits, the enrichment using magnetic particles can increase the detection of trace targeted biomolecules and chemical components with microsensors [9,10,11,12]. 

From all the available technologies to recover the MBs, microfluidics is considered a promising alternative for performing this separation due to its inherent benefits. On the one hand, it allows the integration of the incubation step and the separation process within the same device. Moreover, the use of micrometer-sized devices increases the surface-area-to-volume ratio, which enhances the mass transfer while reducing the use of expensive reagents. Further, a precise control of the fluid flow is possible due to the characteristic laminar flow developed in these devices [13,14,15,16,17,18]. Special attention can also be focused on the capability of separating the beads in continuous mode (i.e., continuous-flow magnetophoresis) as well as the use of permanent magnets as a power-free magnetic field source, which may be important for their implementation as point of care devices in low-resource areas [1,19]. Therefore, the design of such microfluidic-magnetophoretic devices has been the focus of intense research.

A large number of scientific reports have addressed, both theoretically and experimentally, the separation of magnetic materials (either particles or droplets) in continuous-flow channels [20,21,22,23,24]. These studies have analyzed the influence of multiple variables and parameters on the separator performance, such as particle size, flow rates, magnet dimensions and positions, fluid properties, etc. [4,25,26,27,28]. However, a realistic analysis of the geometrical features of microdevices is missing, and literature data on the effect of the geometry of microfluidic channels on particle magnetophoresis are scarce. For instance, Nandy and coworkers identified analytically the relationship between the efficiency of magnetic particle capture in a straight microchannel, that used a line dipole as magnetic source, and several physical parameters, including the channel width [29]. Plouffe et al. assessed the influence of the width of their sheath microfluidic-magnetophoretic device for MCF-7 carcinoma cells separation and concluded that the increase in the channel width negatively affected the capture efficiency [30]. Hale and Darabi reported a computational study for the optimization of a microfluidic-magnetophoretic device for DNA isolation [31]. In their study, the influence of the channel height and the substrate thickness on the magnetic field generated in the device was analyzed. They found that by reducing the substrate thickness and the channel height, a higher magnetic flux was obtained, which contributed to the optimization of the DNA isolation. Nevertheless, these analyses are not realistic, as the channel height, width, and thickness are determined by the employed fabrication methods, and it should be noted that one may influence another, and the cross-sectional area affects the system throughput. Furthermore, we are not aware of the analysis of the cross-sectional shape on particle magnetophoresis, and it is worth mentioning that this is also determined by the fabrication method employed.

In fact, the available fabrication methods have limitations regarding the chip dimensions that could be achieved, the materials that may be employed and/or the geometrical characteristics of the cross section; thus, these factors, as will be shown below, can impact the performance of the system. For example, the etching process utilized for producing glass chips has several limitations regarding the geometrical design due to the isotropicity of the technique, which allows only shallow, semicircular channel cross sections in the glass substrates [32,33,34,35]. As such, the non-symmetrical, U-shaped (U) chip sections derived from wet etching might influence the system performance when compared to channels with the strictly rectangular (R) sections that arise from lithography techniques [36,37,38,39]. Indeed, in our previous work [27], we addressed the separation of magnetic beads from blood solutions in both glass and SU-8 microchannels fabricated with different techniques, and we observed that, even when the same inlet velocities and magnets were applied, the systems rendered different particle recovery. This was attributed to the distinct chips cross sections as well as to the different channel thicknesses (as a result of applying different bonding techniques). Such geometric differences could affect the hydrodynamic and magnetic features of these systems, respectively. However, we did not quantify these effects on the particle recovery or the flow rates that could be effectively employed, since the information regarding the hydrodynamic and magnetic conditions inside the separators was not yet available. Since the goal of a magnetic microfluidic device for bead recovery is to produce a high particle recovery at high throughputs (i.e., at high flow rates of the sample to be treated [16]), the analysis of the performance reported by channels fabricated with different methods and having different cross section shapes, dimensions and thicknesses is an important step in process optimization.

Numerical models provide a powerful platform to further explain, understand, and design the process in advance of the actual, physical fabrication. Although multiple Computational Fluid Dynamics (CFD) models have been applied to predict and optimize particle magnetophoresis in continuous-flow [22], these models have not addressed the analysis of the influence of the cross-sectional dimensions and shapes on the separation results, that would finally determine the optimum fabrication/bonding method. To address these issues, in this work, we perform, for the first time, a realistic analysis of the impact of the chip geometrical features on continuous-flow particle magnetophoresis. More specifically, different Y-Y shaped microfluidic devices are tested by assessing the effect of both channel length and cross-sectional shape and thickness on the separation of magnetic beads for a model biofluid (i.e., blood) and their recovery into an aqueous solution with a permanent magnet. For that purpose, we used our experimentally validated CFD model comprised of a fluidic solver that is coupled to a customized magnetic model [27]. The model is employed to accurately describe the separation of beads inside various channel geometries under different flow rate conditions while keeping invariant the magnet position with regard to the channel surface and the channel width (i.e., the depth is determined by the total chip thickness, which varies with the fabrication/bonding method). Further, dimensionless parameters are developed and introduced to properly compare the different channels and working conditions; these parameters can also be extrapolated to compare other systems different from the ones reported here. Finally, it is noted that this study provides a powerful guideline for the optimization of magnetophoretic applications as well as for the decision-making process regarding the different fabrication techniques for any magnetically-driven separation. 

## 2. Theoretical Methods

### 2.1. CFD Model 

The computational model employed in this work has been presented in our previous publications and has also been experimentally validated [22,27]. Briefly, the model involves an Eulerian-Lagrangian approach to predict the bead movement under the influence of a magnetic field; the beads are considered as discrete elements and their trajectories are computed as follows:

(1) $$m_{p}\frac{dv_{p}}{\mathrm{dt}}=\sum^{\;}F_{\mathrm{ext}}$$

where m_p_ and d**v**_p_/dt are the bead mass and acceleration, and **F**_ext_ is the resultant force vector exerted on each bead. For this analysis, we take into account only the dominant magnetic and hydrodynamic forces acting on the beads.

The magnetic force **F**_m_ is predicted with the “effective” dipole moment method as discussed in our previous work [1], and can be expressed as:

(2) $$F_{m}=\mu_{0}V_{p}f\left(H_{a}\right)\left(H_{a}\cdot \nabla \right)H_{a}$$

where:

(3) $$\begin{array}{c} f\left(H_{a}\right)=\chi_{p,e}\;\;\;\;\;\;\;\;\;\;\;\;\;\;\forall \;\;\;\;\;\;\;\;\;\;\;\;\left|H_{a}\right|<\left(\frac{\left(x_{p}-x_{f}\right)+3}{3\left(x_{p}-x_{f}\right)}\right) \end{array}M_{s,p}$$

(4) $$f\left(H_{a}\right)=\frac{{\;M}_{s,p}}{\left|H_{a}\right|}\;\;\;\;\;\;\;\;\;\;\;\;\;\;\forall \;\;\;\;\;\;\;\;\;\;\;\;\left|H_{a}\right|\geq \left(\frac{\left(x_{p}-x_{f}\right)+3}{3\left(x_{p}-x_{f}\right)}\right)M_{s,p}$$

where µ_0_ is the permeability of the free space (4π·10^−7^ H·m^−1^), Vp is the particle volume, **H**_a_ is the applied magnetic field at the bead center, χ_p_ and χ_f_ represent the magnetic susceptibilities of the bead and the fluid, respectively. χ_p,e_ and M_s,p_ are the effective susceptibility of the bead (that can be related to the intrinsic susceptibility, χ_p_) [40] and the particle saturation magnetization. The particles are assumed spherical with a diameter of 4.9 µm and a density equal to 2000 kg·m^−3^. The saturation magnetization and susceptibility values employed are M_s,p_ = 1.5 × 10^4^ A·m^−1^ and χ_p,e_ = 0.25, which are within the range of magnetization and susceptibilities reported for commercial beads [27,41]. The surrounding fluids are considered non-magnetic due to their low susceptibilities in comparison to the beads. In order to calculate the magnetic field distribution inside the microdevice, we solved the analytical model developed by Furlani [42]. A permanent magnet of dimensions 10 × 5 × 3 mm^3^ is modeled as the magnetic field source, which is commercially available [27]. This magnet generates a magnetic field of approximately 500 mT in its pole surface.

The hydrodynamic force **F**_hd_ is numerically predicted in this work using the following expression:

(5) $$F_{\mathrm{hd}}=-V_{p}\nabla P+M_{\mathrm{added}}\left(\frac{\partial v}{\partial t}-\frac{\partial v_{p}}{\partial t}\right)+F_{\mathrm{drag}}$$

where:

(6) $$F_{\mathrm{drag}}=\frac{1}{2}\rho \left(v-v_{p}\right)\left|v-v_{p}\right|A_{p}C_{d}$$

and:

(7) CD=24Rep+61+Rep+0.4

where P is the pressure and M_added_ is the added mass, equal to 0.5ρV_p_, being ρ the fluid density [22]. **v** is the fluid velocity, **F**_drag_ is the drag force and A_P_ is the bead cross sectional area, which can be written as A_P_ = πr_p_^2^, being r_p_ the particle radius. C_D_ is the drag coefficient for steady-state flow around a sphere [43] and Re_p_ is the particle Reynolds number [27,44]. 

To evaluate **F**_hd_, the properties of the fluids as well as the velocity profile are needed. One of the fluids in our analysis, blood, presents a non-Newtonian rheology. However, we modeled it as a Newtonian fluid and its viscosity was quantified with an analytical, empirical expression, and has a value of 3.5 cP [45,46]. It should be noted that it has been demonstrated in previous work [47] that blood follows a Newtonian rheology when the shear rate exceeds about 100 s^−1^, and we have also experimentally validated this assumption [27]. On the other hand, the aqueous buffer solution was modeled as water with a constant viscosity equal to 1 cP. 

As for the fluid velocity field, we employed the Navier Stokes and continuity equations for incompressible flows, accounting for the effect of the bead motion on the fluid flow through a two-way coupling model:

(8) $$\frac{d\left(\rho v\right)}{\mathrm{dt}}=-\nabla P+\mathrm{div}\left(\tau \right)+\frac{1}{V}F_{P}$$

(9) ∇·(v)=0

where the div(**τ**) term denotes the contribution of shear stress on the fluid velocity. 

The last term in Equation (8) represents particle induced fluid accelerations, and **F**_P_ can be written as:

(10) $$F_{P}=-\sum^{\;}\left[F_{\mathrm{drag}}+M_{\mathrm{added}}\left(\frac{dv}{\mathrm{dt}}-\frac{dv_{p}}{\mathrm{dt}}\right)\right]$$

### 2.2. Geometric Configurations and Simulation Setup

**Figure 1:**
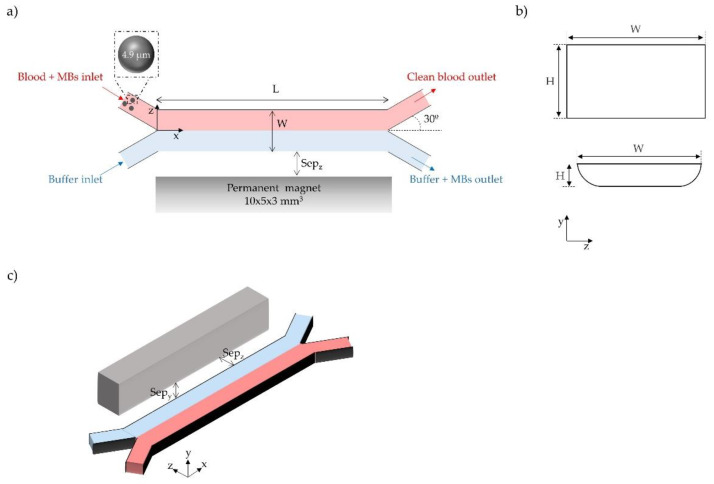
(**a**) Top view of the microfluidic-magnetophoretic device, (**b**) Schematic representation of the channel cross-sections studied in this work, and (**c**) the magnet position relative to the channel location (Sep_y_ and Sep_z_ are the magnet separation distances in y and z, respectively).

We analyzed the effect of the cross-sectional shape and channel length/thickness on the device performance (i.e., bead capture and system throughput), by studying two different cross sections with different thicknesses and depths and three channel lengths. All the devices have two inlets and two outlets with a Y-Y shape, as presented in Figure 1a. The length (L) of the devices was varied from 2 to 10 mm. The cross sections are displayed in Figure 1b, where H represents the depth of the channel while W is the width, which is similar for all tested geometries. As seen, channels with rectangular and U-shaped cross sections have been considered, as these are the typically derived from the most popular fabrication methods [13,27,48,49]. Furthermore, the magnet location relative to the channel for all of the studied systems is illustrated in Figure 1c.

In Table 1, the dimensions of the microchannels under study are listed. It should be noted that, when wet etching techniques are employed for fabrication, microchannels with U shapes are obtained and the minimum channel width (W_min_) determines H, according to the following expression [50,51]:

(11) $$W_{\min}=x+2\;H$$

where x denotes the etching mask opening width, usually 20 µm.

For our devices, the width of the inlets/outlets is the narrowest (approximately half of the main channel width), and these inlets/outlets determine the H value that can be achieved by wet etching, which in this case is only 60 µm as seen in Table 1. On the other hand, the depth of rectangular channels is limited by the thickness of the SU-8 layer that can be produced, since thick films could lead to difficulties in the fabrication process and subsequently to faults in the final microchannels. Common SU-8 layer thickness ranges from 2 to 260 µm, depending on the SU-8 type and on several fabrication variables [52,53,54,55]. Since in our previous work [27] 200 µm depth R devices were used to experimentally validate our CFD model, and this depth is within the range of feasible SU-8 film thicknesses, in this work we set the depth of rectangular channels in 200 µm as well. All of these limitations are important as they affect the hydraulic diameter (D_h_) and the chip volume. In order to suitably compare rectangular and U-shaped cross sections, we also calculated the D_h_ as well as the chip volume by using the appropriate equations for each type of device [56,57], which are detailed in Table 1. Rectangular and U-shaped devices were further characterized and compared by estimating the fluidic resistance (R_f_) of the channels according to Equation (12) [58,59,60]:

(12) $$R_{f}=\frac{8\;\eta \;L}{{\pi \;R}_{h}^{4}}$$

where η is the fluid viscosity and R_h_ the hydraulic radius of the channel. 

The R_f_ value for each of the studied geometries is listed in Table 1. By taking all of the previously described parameters into account, we can perform a realistic analysis of the geometrical features of the chip.

**Figure 2:**
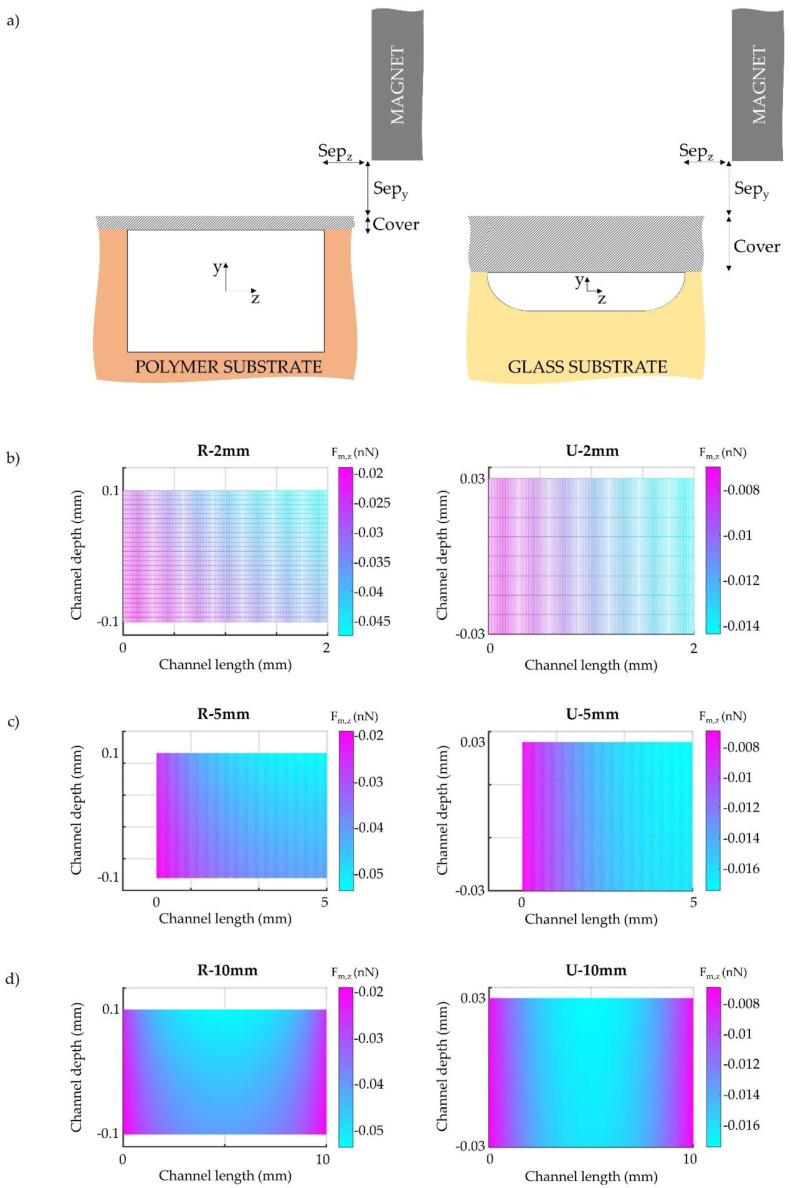
(**a**) Channel-magnet configuration and (**b**–**d**) magnetic force distribution in the channel midplane for 2 mm, 5 mm and 10 mm long rectangular (left) and U-shaped (right) devices.

The technique and the material selected for the device fabrication not only influence the channel geometry but also determine the thickness of the total chip. During the fabrication process, a bonding step is necessary, where a slide is placed on top of the channel sealing the device. As such, the thickness of the cover bonded to the channel affects the channel-magnet distance, and thus, the magnitude of the magnetic force acting on the beads. For glass channel configurations, 1 mm thick microscope slides are generally employed as covers, whereas polymer devices can be sealed with submillimeter cover plates [52,55,61,62,63]. In our previous work [13,27], we experimentally employed devices with cover thicknesses of 80 µm and 1 mm for polymer and glass channels, respectively. Since these values are in agreement with the cover slide thicknesses considered in the literature for fabricating polymer and glass channels, we considered the same dimensions in this work. In Figure 2a, the magnet position relative to each device is depicted. For all of the systems, the magnet-channel distance in z-direction (Sep_z_) is 190 µm. Correspondingly, Sep_y_, is defined as the separation between the magnet and the surface of the device (i.e., surface of the channel slide) in y-direction. This dimension was set to 1 mm for both types of devices. However, the total magnet-channel separation in y-direction should also account for the thickness of the cover slide, which was different for each configuration, as previously mentioned. In this case, the cover dimension in y is greater for the U device, as presented in Figure 2a. Although Sep_z_ and Sep_y_ take the same values in this work for the two cross section shapes studied, the cover thickness affects the magnetic field generated inside the device, and thus, the magnetic force acting on the beads. Therefore, in Figure 2b–d, we have also presented the magnetic force distribution in x and y directions inside both channels for the three tested lengths. To plot this distribution, we simulated the force acting on the particles that are located at the channel midplane, z = 0. Inspection of this plot indicated that, although the chip-magnet separation remains the same for both devices (same Sep_z_ and Sep_y_), the thickness of the cover materials relative to, and combined with the depth of the channel, affects the magnitude of the magnetic force developed across the channel, impacting the performance of the separation. Thus, thick cover slides are not desirable when they are placed between the magnet and the particles to be separated. 

In this case, the average magnetic force in z direction (the direction of the magnetic field gradient) is estimated to be 0.038 nN for R devices, whereas it decreases to approximately 0.013 nN for U devices. While there is this difference between the R and U devices, Figure 2b–d indicate that the average magnetic force acting on the particles does not dramatically change for each cross section shape; both the magnetic field and the magnetic field gradient are similar along L for this channel-magnet configuration. It should be noted that relatively thin slides (polymer devices) are not appropriate for processes where high pressures or temperatures are required; for these cases, the mechanical stability of the device (influenced by the hardness and thickness of the cover as well as the bonding method) is paramount.

In summary, to elucidate the effect of the geometry on the device performance, channels with different cross section shapes were analyzed. The width and the three tested lengths of these channels, as well as the channel-magnet position were similar for both types of cross section devices, whereas the depth (and thus the volume) and the cover thickness, which are highly fabrication-dependent variables, change with the cross section shape. These differences create different hydrodynamic and magnetic conditions inside the channels, which we demonstrate below ultimately determine the device performance.

Regarding the simulation conditions, we initially solved the flow field and particle magnetophoresis using a 3D model to evaluate the influence of the cross section on the velocity distribution and on the particle locations across the cross section, but due to the increased computational cost of these simulations, the influence of the channel dimensions on the magnetophoresis studies was simulated in 2D (although the magnetic force was calculated analytically in 3D). A uniform grid was employed for all the simulations; the mesh was composed of approximately 400,000 cells.

For studying the magnetophoresis process under different flow conditions, the inlet flow rate was varied between 0.005 and 3 µL·s^−1^ depending on the geometry. These hydrodynamic conditions ensure medium shear rates in the channels, under which blood can be treated as a Newtonian fluid. Nevertheless, shear stresses are still too low to observe hemolysis [64,65]. The calculated average velocity for every flow rate was used as an initial condition. As for the boundary conditions, a no slip condition (zero velocity) was applied along the microchannel walls while at the outlet, we used a calculated outflow boundary. Beads were introduced into the domain at a constant concentration of 0.1 g·L^−1^, which corresponds to a flow of 10–2500 particles·s^−1^, depending on the fluid flow rate; they were randomly injected through the cross section of the blood inlet as seen in Figure 1a, with a velocity equal to the blood stream. The simulation time was kept below 15 s for all the cases, which allowed us to track the path of 100–400 beads.

The model was solved using the commercial CFD software FLOW-3D (version 11.2, Flow Science, Inc., Santa Fe, NM, USA). The magnetic force and the magnetic field distribution due to the magnet were calculated in a customized FORTRAN subroutine compiled in Visual Studio 2013 (Microsoft, Redmond, WA, USA). MATLAB (version 2015, The MathWorks, Inc., Natick, MA, USA) was also employed for the visualization of the magnetic field and force distributions inside the different channels. The simulations were performed on a 48-core workstation with 128 GB of RAM.

### 2.3. Dimensionless Analysis

Two dimensionless parameters were introduced to elucidate the effect of the channel geometry (cross section shape and dimension, cover thickness, device length and volume) on the system performance and to efficiently compare across the broad set of geometries. The first parameter, J, relates the magnetic and the drag forces that are exerted on the beads in z and x directions, respectively. With this parameter, the fluidic and magnetic variables and parameters that influence the bead trajectory (particle volume, magnetization, magnetic field strength and gradient inside the channel, viscosity of the fluids and inlet mean velocities) are considered. The J parameter can be described as [66]:

(13) $$J=\frac{\bar{F_{m,z}}}{\bar{F_{\mathrm{drag},x}}}=\frac{\mu_{0}V_{p}f\left(H_{a}\right)\left(H_{a,z\left(\frac{L}{2},\;\frac{H}{2}\right)}\cdot \nabla \right)H_{a,z}}{6{\pi r}_{p}\eta_{b}{\;\bar{v}}_{b}}$$

To calculate J, the magnetic force was estimated at the midplane of the channel $\left(\frac{L}{2},\;\frac{H}{2}\right)$ for each channel geometry. For determining the drag force, the viscosity ($\eta_{b}$) and average velocity (${\bar{v}}_{b}$) of the blood phase were considered, since successful particle recovery entails the migration of the beads from the blood, where they are initially, to the buffer stream.

The second parameter, θ, balances both the residence time of the particles in the microdevice (t_res_) and the time they require to travel from the blood to the buffer solution considering that they move completely perpendicular to the flow direction (t_m_) [48,67]. Apart from the variables and parameters included in J, the channel length (L)/width-depth (D_h_) ratio, defined as the aspect ratio, is included in θ. Hence, this design parameter can be written as:

(14) $$\theta =\frac{t_{\mathrm{res}}}{t_{m}}=\frac{L}{D_{h}}\cdot J$$

In this way, J can be used for studying the performance of a single device through the analysis of the fluidic and magnetic conditions developed inside the chip, accounting for the overall magnet-channel distance, and hence including the effect of the different cover thicknesses considered. Conversely, θ takes into account the geometrical characteristics of the device (width, depth, length, and thus, volume), which allows us to compare the performance of channels with different size and shape.

In order to assess the system performance, for this study the bead recovery is defined as the percentage of particles that leave the system through the buffer outlet compared to the total number that leaves the system (i.e., bead recovery is normalized to the outlet):

(15) $$\mathrm{Bead}\;\mathrm{recovery}\;\left(\mathrm{BR}\right)=\frac{{\mathrm{Particles}}_{\mathrm{buffer}}}{{\mathrm{Particles}}_{\mathrm{blood}}+{\;\mathrm{Particles}}_{\mathrm{buffer}}}\cdot 100$$

## 3. Results and Discussion

In this section, the effect of the microchannel geometry on both particle recovery and system throughput (i.e., blood treated flow rate) is explored. First, the cross section shape (keeping the same width, W) and the channel length will be evaluated, while the global analysis of the system taking into account the dimensionless parameters will be presented subsequently. 

### 3.1. Influence of Microchannel Cross Section Shape

**Figure 3:**
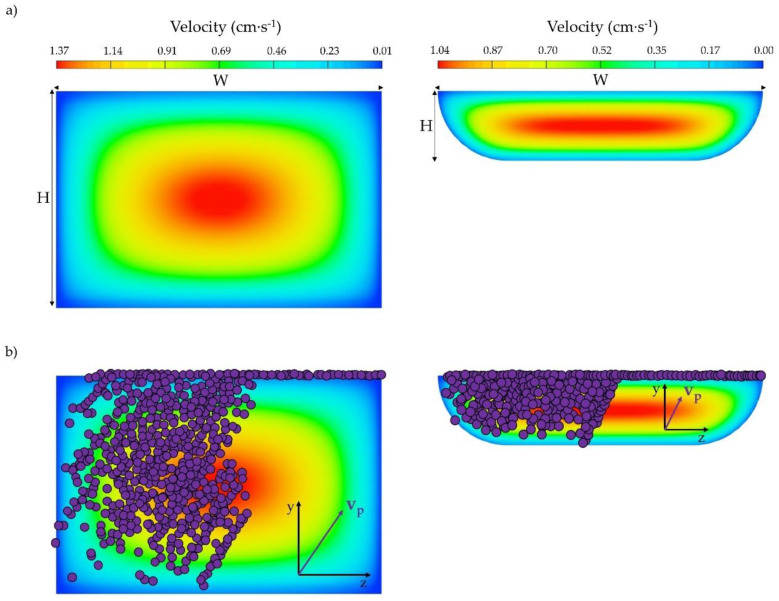
(**a**) Velocity distribution in a section perpendicular to the flow for rectangular (left) and U-shaped (right) cross section channels, and (**b**) particle location in these cross sections.

As mentioned before, the cross section shape may impact the velocity profile developed inside the microfluidic channel, and thus, the flow rates that could be employed to obtain complete recoveries. In order to assess the cross section effect, the velocity field achieved inside different channels was simulated. In Figure 3, the velocity distribution developed in a section perpendicular to the flow and the resulting bead positions in the channel cross section (for all x coordinates) are presented for devices with U-shape and rectangular cross sections when the same average velocity (0.86 cm·s^−1^) is applied at the inlets.

It can be perceived that, although the magnitude of the maximum velocity is lower for the U channel, the area over which this maximum velocity expands is higher in comparison to the rectangular channel. In fact, for the rectangular geometry, the area where the velocity achieves the highest value is lower and only located at the center of the cross section. Consequently, beads are exposed to the maximum velocity to a greater extent in U channels than in rectangular ones, as readily observed in Figure 3a. Nevertheless, since the maximum velocity achieved inside U channels is lower than the maximum velocity value in R channels, similar drag forces could be expected when applying the same inlet velocity to both channels. This different velocity distribution along with a different magnetic force field obtained inside the channels could give rise to a dissimilarity in the force balance acting on the particles.

We have simulated the particle locations across the cross sections of the channels for this working condition; the results are presented in Figure 3b. It can be observed that the particles move from the blood phase (left) to the right (buffer), and from the bottom wall to the top of the channel (where the magnet is placed) for both devices. However, the qualitative z component of the particle velocity vector **v**_p_ (flow into the figures) has a greater magnitude for the R channel, implying that, higher recoveries can be achieved within the R device. Thus, in rectangular channels the operating conditions acting on the beads seem to be more favorable for particle magnetophoresis than in U-shaped devices. In other words, for obtaining the same particle capture, the inlet flow rate in U-shaped channels must be reduced.

**Figure 4:**
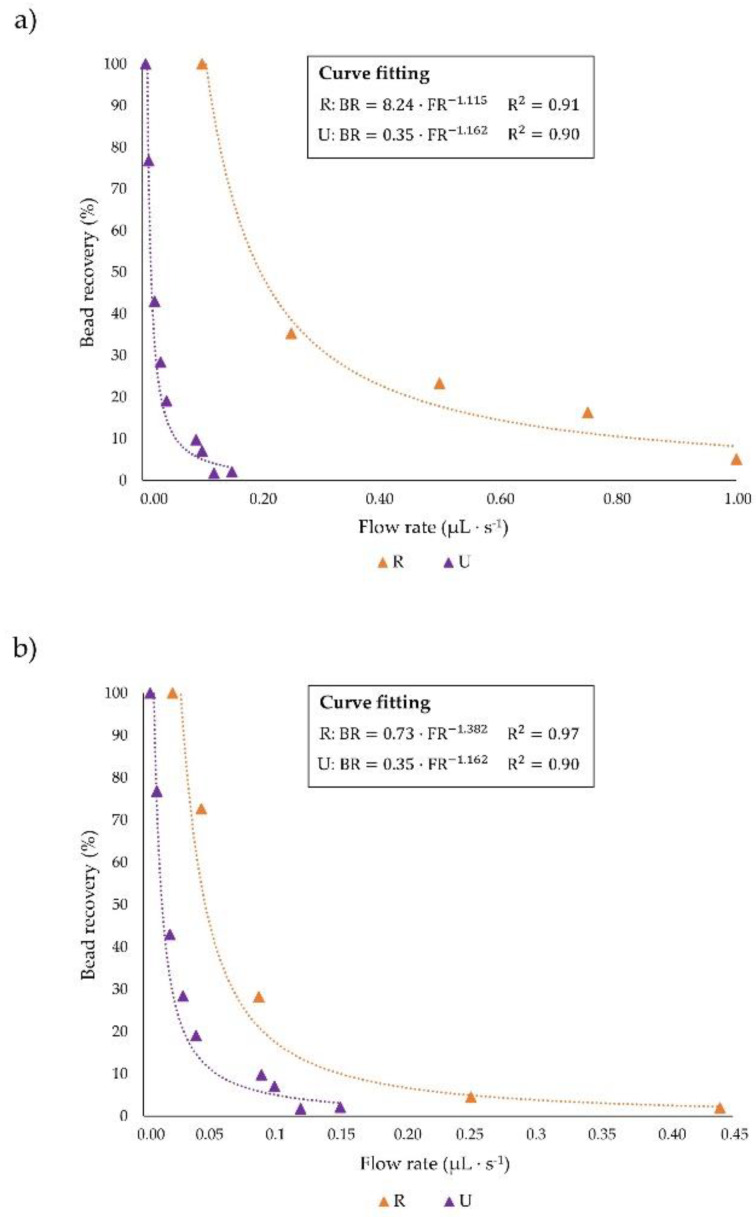
Influence of fluid flow rate on particle recovery when the applied magnetic force is (**a**) different and (**b**) equal in U-shaped and rectangular cross section microdevices.

We have calculated the bead recovery as a function of the fluid flow rate for both channels. The results are presented in Figure 4a, where the equations of the least square curve fitting are specified; BR corresponding to the percentage of bead recovery and FR corresponding to the flow rate. It can be observed that, for attaining comparable recoveries, the flow rate needs to be reduced at least one order of magnitude for the U channel in comparison to the rectangular one, which results in an undesirable decrease of the system throughput, and thus in an increase of the time required for the separation and concentration of the target compounds. This is attributed to the lower cross sectional area of the U device in comparison to the R device when comparable widths are employed (i.e., the cross sectional area of U channels is limited by the width as shown in Equation (11)), and also to the presence of a thicker cover slide, which affects the magnitude of the magnetic force. 

In order to elucidate the effect of the cross section shape on the device performance, simulations applying the same magnetic field and inlet fluid velocity were carried out for rectangular and U-shaped channels. For achieving comparable magnetic forces, the same cover thickness (1 mm) was considered in both devices.

Figure 4b displays the dependence of the particle capture with the inlet flow rate when similar magnetic and drag forces are applied. It can be deduced that although comparable magnetic and fluidic conditions were ensured, rectangular channels enable the treatment of flow rates up to four times higher than U-shaped ones while fulfilling analogous recoveries. It is worth mentioning that for designing magnetophoretic microseparators, the required bead collection, as well as the sample volume to be processed during a specific time (i.e., flow rate) must be established. However, we also determined the influence of the fluid velocity on the particle capture (Figure S1); these results show that, when the particle recovery is presented as a function of the average inlet velocity, comparable bead collections are achieved in both devices, which is expected since similar J values are derived for both channels. This observation led us to conclude that the hydrodynamic conditions inside the channels are not highly affected by the cross section shape. Thereby, the weaken performance of U-shaped channels is expected to be due to their lower cross section area derived from the limitations to fabricate deeper glass channels. Nevertheless, there are some situations where glass channels might be preferred over SU-8 or polymeric devices on account of their strength, durability, chemical and heat resistance and optical transparency [33,34,35,68]. In this regard, optimizing their performance in order to enhance the system throughput while maintaining complete bead collection is imperative so as to microfluidic-magnetophoretic devices could be effectively employed. Therefore, in the following sections, the influence of the microchannel geometry (i.e., length and volume) and the process optimization are addressed.

### 3.2. Influence of Microchannel Length

The influence of microchannel length on the system performance was assessed by analyzing three channel lengths (L = 2, 5 and 10 mm) for both rectangular and U-shaped section channels. Specifically, only the channel length was modified for this study whereas the channel cross sectional dimensions were not modified (see Table 1). As previously demonstrated, the average magnetic force exerted on the beads is not significantly influenced by the chip length for each cross section shape. Hence, the average **F**_m_ for the three tested lengths is approximately 0.013 nN in the U-shaped and 0.038 nN in rectangular devices.

**Figure 5:**
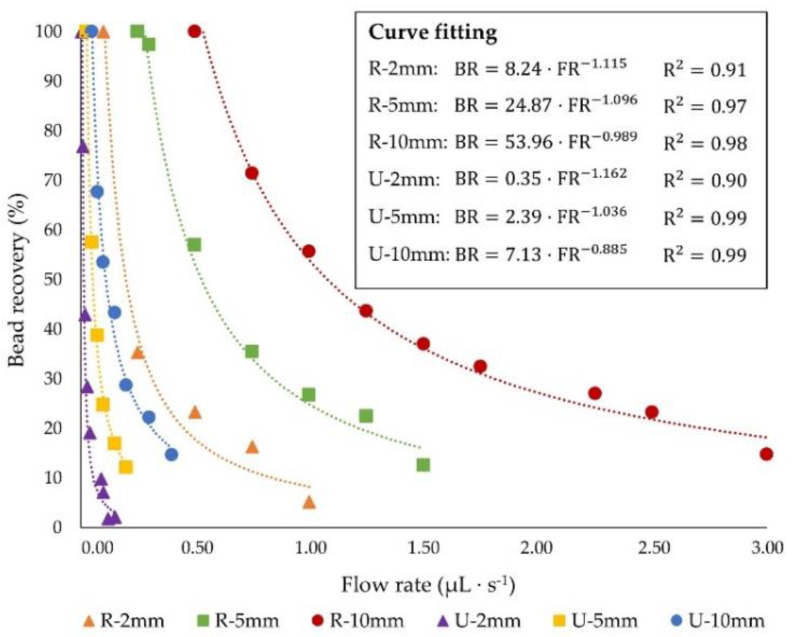
Magnetic bead capture as a function of fluid flow rate for all of the studied geometries.

Therefore, we examined the range of flow rates that can be applied to achieve complete bead recovery, by accounting for the effect of channel elongation. In Figure 5, the particle capture under variable fluid flow rates is depicted for the three lengths and the two cross sectional shapes examined. It can be seen that longer channels deliver greater residence time to the particles to go along the z-direction of the device for the same flow rate, promoting their collection at the buffer outlet (Figure S2). As such, for equal flow rates, a five-fold increase of channel length leads to an enhancement of the particle recovery of about 65% for rectangular and 44% for U-shaped section devices (Figure S3). This means that increasing the channel lengths by a factor of 5 did not result in equal recovery improvements between the R and U geometries. This disagreement between both systems is also attributed to the different cross sectional area as well as to the dissimilarity in the magnetic force achieved inside the chips. The improved performance of rectangular channels also stems from their lower R_f_ values, which are one or two orders of magnitude lower than those observed for U-shaped devices with the same length. Comparing channels with R_f_ values of the same order of magnitude, that is 10 mm long rectangular and 2 mm long U-shaped chips, it can be noticed that the R-10mm device allows the management of flow rates 100 times higher than U-2 mm while attaining the entire bead recovery, which implies a significant enhancement in the system throughput.

Furthermore, Figure 5 reveals that the 10 mm long chip with rectangular section offers the best performance, since it provides the complete magnetic capture while treating sample flow rates one or two orders of magnitude higher than the other geometrical configurations, improving the system throughput. 

Once the cross section shape and channel length have been individually analyzed, in the next section the overall optimization of the system will be presented along with useful guidelines regarding the channel design.

### 3.3. Dimensionless Channel Design

**Figure 6:**
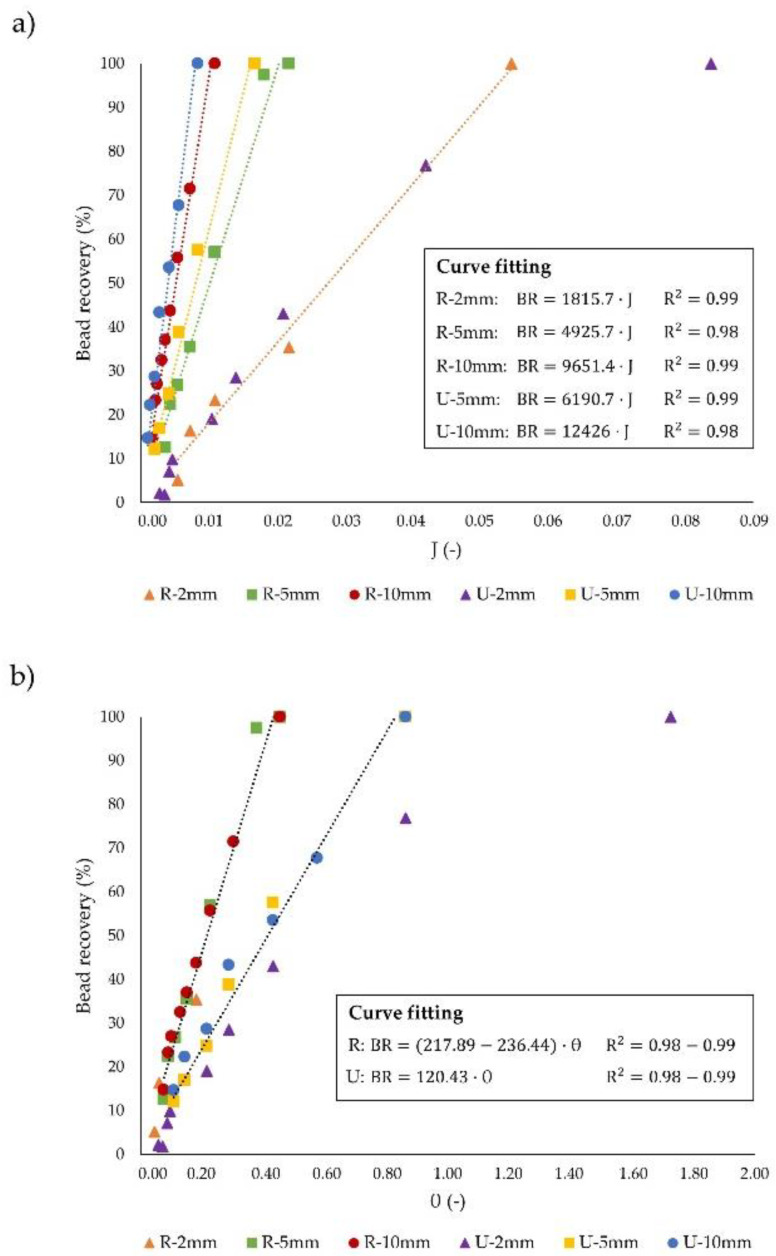
Influence of (**a**) magnetic and fluidic forces (J parameter) and (**b**) channel geometry (θ parameter) on particle recovery. Note that U-2mm does not accurately fit a line.

In this subsection, we apply the two dimensionless design parameters, J and θ introduced above, to compare all the geometries under study and characterize their geometrical features. The J parameter is used to consider the operating regime of the microseparators into two regimes: hydrodynamic J < 1, and magnetic J > 1, as illustrated in Figure 6a. It can be easily deduced that for both devices, the particle recovery increases with J; in this case, J increases due to a decrease in the flow rate since the **F**_m_ is fixed for each cross section shape. Furthermore, complete particle capture is achieved working in hydrodynamic conditions (i.e., drag forces larger than magnetic ones) for all the systems (J_2_^R^ = 0.054; J_5_^R^ = 0.022; J_10_^R^ = 0.011; J_2_^U^ = 0.084; J_5_^U^ = 0.017; J_10_^U^ = 0.008). In addition, we found that the J value that corresponds to complete particle recovery is similar for both devices; as seen in Figure 6, the recovery as a function of J is dependent on L but independent on the cross sectional shape. Since the J parameter scales directly proportional to the magnetic and inversely to the drag force (Equation (13)), similar J values for each channel length stem from the compensation of the higher values of **F**_m_ and **F**_drag_ for rectangular channels in comparison to the U-shaped. Additionally, due to the similarity of the magnetic force exerted on the particles regardless the channel length for each cross section shape, the J parameter necessary to achieve complete recovery decreases as the channel length is increased. This is due to the fact that, by using longer devices, the applied flow rate can be also increased, and thus the fluid velocity inside the channels, which leads to higher drag forces (Figure S2). Therefore, optimum systems will be those that allow complete bead recovery at the smallest J, since the throughput could be enhanced up to 5 times by working with 10 mm R (J_10_^R^ = 0.011) compared to 2 mm R (J_2_^R^ = 0.054) channels, or 10 times when 10 mm U (J_10_^U^ = 0.008) devices are used instead of 2 mm U (J_2_^U^ = 0.084).

Following the dimensionless analysis, to assess the overall influence of the channel geometry, the dimensionless parameter θ was exploited. In Figure 6b, the percentage of particle recovery as a function of θ for all the geometries is shown. As occurs with J, particle recovery is favored by increasing θ. However, contrary to what happened with J, the particle recovery as a function of θ is not dependent on the length of the channel, but slightly changes with the shape of the cross section. Accordingly, we found that, for the two types of cross sections considered in this study, the effect of increasing the L/D_h_ ratio is compensated with the decrease in the J parameter, due to the rise of the fluid drag force which results from the larger velocities that could be applied to obtain the same bead collection. For this reason, particle recovery as a function of θ does not change with the channel length. Thereby, regardless the aspect ratio, with rectangular section channels complete recoveries are accomplished approximately 2 times faster in comparison to U-shaped ones, as derived from the linear relationship (R^2^ ≈ 0.98) between the θ parameter and the percentage of particle capture for all the geometries except for the 2 mm long U device. This fact leads to lower θ values for rectangular channels (θ^R^ = 0.45) than for U-shaped ones (θ^U^ = 1.73) for attaining complete particle capture. Furthermore, for comparable aspect ratios, such as 5 mm long U (L/D_h_ = 51) and 10 mm long R (L/D_h_ = 42) or 2 mm long U (L/D_h_ = 21) and 5 mm long R (L/D_h_ = 21), the values of the J parameter that yield entire capture are lower for rectangular channels (J_5_^U^ = 0.017; J_10_^R^ = 0.011; J_2_^U^ =0.084; J_5_^R^ = 0.022), and hence lower θ. It should be noted that because of the limitations for producing deeper glass devices, the cross section area of U is lower than for rectangular channels, since the limitations regarding the channel depth in R chips are less restrictive. Thus, similar aspect ratios entail longer R than U channels. Consequently, the higher magnetic forces and residence time combined with the different velocity distribution of rectangular channels make possible the use of higher velocities (i.e., higher drag forces) than in U-shaped, thus reinforcing the evidence that rectangular sections promote the improvement of the system throughput. As such, systems that bear complete recoveries with the lowest θ are preferred. 

On the other hand, the comprehensive evaluation of the channel geometry has also been assessed by examining the impact of the channel volume on both the magnetic capture and the system throughput for each independent geometry.

**Figure 7:**
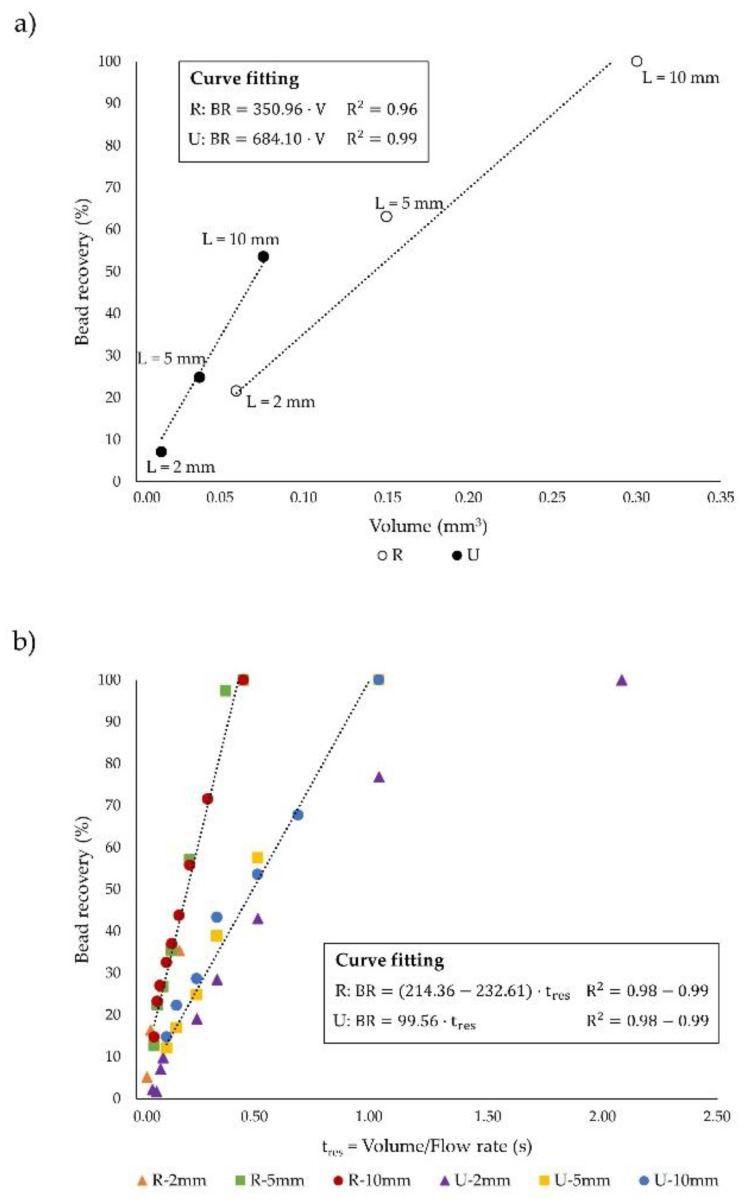
Dependence of bead capture on the (**a**) functional channel volume and (**b**) particle residence time (t_res_). Note that in the curve fitting expressions V represents the functional channel volume and that U-2mm does not accurately fit a line.

For that purpose, Figure 7a illustrates the bead recovery percentage as a function of the functional channel volume (without taking into account the substrate and cover volumes presented in Figure 2a) when the same inlet velocity is considered (1.92 cm·s^−1^). It should be mentioned that the functional channel volume corresponds to the branch of the Y-Y shaped device whereby blood flows; since the microdevices considered in this study are symmetric in the z-direction (see Figure 1a), the total system volume is twice the channel volume. As presented in Figure 7a, by increasing the channel volume (microchannel lengthening), the particle residence time in the chip also increases, which promotes their collection at the buffer channel outlet (Figure S2). Furthermore, the analysis of the functional volume of the channels reveals that U devices are more efficient since they deliver higher recoveries than R devices when comparing the same volume. In this regard, for having comparable channel volumes, the length of U chips must be five times higher than for rectangular, due to the smaller cross section shape of U devices that arises from the weaknesses of the fabrication method as previously stated. Under these conditions, U-shaped chips provide higher residence time to the particles, which fosters their capture, thus leading to higher recoveries than in rectangular devices, although the magnetic force exerted in the rectangular channel is higher. This means that the effect of channel elongation has a greater impact than the application of higher magnetic forces. Similarly, for attaining comparable bead recoveries, the length of U channels should be approximately 2.5 times higher than the length of R channels. However, due to the lower deepness of U-shaped channels in comparison to rectangular, the volume of U is lower than of R devices, which demonstrates the more efficient performance of high aspect ratio (L/D_h_) channels in terms of bead capture, given that they render similar recoveries with lower channel volumes. 

Despite this efficient performance, the effect of the flow rate that can be treated has not been considered in Figure 7a. Since the fulfillment of the design requirements (i.e., treating relatively high blood flow rates while entirely capturing the beads) for a realistic application of these magnetophoretic microseparators is intrinsically related to the channel geometry, and hence to the chip volume, in Figure 7b the dependence of the bead recovery with the ratio between the functional channel volume and the treated blood flow rate (namely residence time) is displayed. It can be noticed that such relationship is not influenced by the channel length for each cross section shape. Nevertheless, the residence time of the particles in the device required for attaining similar recoveries is lower in rectangular than in U-shaped channels; thus it is possible to achieve the complete bead capture by applying up to five times higher flow rates in R than in U, having both types of devices the same volume (Figure S2). Therefore, we can conclude that while U-shaped chips are more efficient in terms of the lower volume they require for a desired recovery, rectangular channels allow higher sample flow rates to be subjected to magnetic forces for achieving the specified recovery with the same channel volume.

Overall, the results of the global analysis carried out in this work are presented in Table 2. As seen in this table, for the same fluidic conditions, the higher magnetic force acting on rectangular devices yields greater J values, which results in a higher bead collection in rectangular devices. Additionally, for similar θ values the recoveries obtained with rectangular channels can be approximately 3 times higher than with U-shaped, hence, displaying a faster raise in the percentage of particle capture with θ. This different dependency of the particle recovery on θ implies that particle capture attained by using rectangular in contrast to U-shaped section channels can be increased as much as 14 times. In this regard, with 10 mm long R channels obtaining complete recoveries is possible at inlet fluid velocities of 1.92 cm·s^−1^, whereas U-shaped devices must be elongated to a greater extent (or several channels in series must be used) in order to increase the residence time of the beads in the device and thus promote their entire recovery (Figure S2). In other words, the particle residence time in U channels should be approximately twice than that in rectangular ones for achieving similar recoveries in both types of channels when applying the same inlet velocity; thus, rectangular devices allow attaining complete particle capture while treating blood flow rates 4.4 times higher than in U channels. Due to the higher residence time required by U-shaped chips, several devices have to be arranged in parallel for concurrently delivering comparable recoveries and throughputs to rectangular channels. Accordingly, for successfully treating a flow rate of 1 mL of blood per hour while capturing entirely the beads, one 10 mm long R device or six 10 mm long U devices, operating in parallel, are required. However, it should be taken into account that the use of several microseparators either in series or in parallel, which is needed so that the performance of U devices is similar to that of rectangular ones, may lead to higher fabrication and operational costs, thus negatively affecting the system efficiency.

## 4. Conclusions

The separation and concentration of targeted analytes from complex mixtures are key to enhance their subsequent detection or analysis. This pre-separation/concentration stage can be carried out in microfluidic magnetophoretic platforms, where the potential advantages of both magnetic beads and microfluidics are exploited. Nevertheless, for being successfully used, these devices should deliver high particle-analyte complex recovery at high sample flow rates, thus, reducing the time required in the stage prior to the detection or analysis.

In this work, we have addressed the optimization of the geometry of Y-Y shaped microchannels for carrying out the continuous-flow particle magnetophoresis from biofluids. More specifically, for microdevices with the same width, we evaluated the influence of the channel length/thickness and cross section shape on the system performance (i.e., particle recovery capacity and throughput expressed as residence time). To effectively compare all the systems under study, we have made use of two dimensionless parameters, which account for the forces acting on the beads (J) and for the coupling of these forces and the geometrical features of the channels (θ). 

According to our findings, the lower magnetic force, due to the higher channel thickness, in addition to the lower cross sectional area as well as a different velocity distribution developed inside U channels lead to a notable reduction of the flow rates that can be treated for attaining complete particle recoveries in comparison to rectangular section channels, lessening the system throughput. Hence, for each specific cross section shape, we dealt with the improvement of the microseparators performance by lengthening the channels. As expected, the resulting increase of the particles residence time in the device promotes their complete capture at higher flow rates, thus, significantly improving the system throughput. This means that channel elongation favors the system operation in a hydrodynamic regime, i.e., J parameter values considerably lower than 1. We have also elucidated that the bead collection as a function of J only depends on the channel length, whereas the different relationship between particle recovery and θ for the tested geometries is determined by the cross section shape. Thereby, rectangular channels accomplish complete recoveries faster than U-shaped section devices, being possible working with θ values approximately 4 times lower when rectangular channels are used. Finally, the effect of the system volume was analyzed, finding that for the same device volume, higher recoveries were obtained with U-shaped section channels. Nevertheless, a global analysis of all the geometries considered in this work allows one to conclude that the main weakness of U devices is their low throughput for achieving the desired capture in comparison to rectangular ones; hence, rectangular section channels are the best alternative since they need shorter residence times for a specific recovery than U devices. Despite the noteworthy differences in the performance between devices with these cross sectional shapes, U-shaped chips are imperative for applications where the use of glass channels is required due to the working conditions or the fluid nature. Therefore, we consider that the design guidelines provided in this systematic study promote the understanding of the importance of channel geometrical features on the system performance. Additionally, they prove potentially valuable to tackle the rational design of any magnetophoretic microseparator, extending their exploitation beyond the geometries considered in this work. 

## Figures and Tables

**Table 1:** Dimensions and geometric features of the microchannels under study.

	Rectangular Shape (R)			U Shape (U)		
L (mm)	2	5	10	2	5	10
W (µm)	300	300	300	280	280	280
H (µm)	200	200	200	60	60	60
D_h_ (µm)	240	240	240	97	97	97
L/D_h_	8	21	42	21	51	103
Volume (mm^3^)	0.12	0.3	0.6	0.03	0.08	0.15
R_f_ (10^12^ Pa·s·m^−3^)	0.33	0.83	1.65	6.46	16.14	32.29

**Table 2:** Comparative analysis of all channel configurations for the same inlet fluid velocity (1.92 cm·s^−1^).

	Particle Recovery (%)	Throughput (µL·s^−1^)	J (-)	Θ (-)	t_res_ (s)
U-2 mm	7.09	0.1	0.004	0.086	0.1
U-5 mm	24.78	0.1	0.004	0.216	0.26
U-10 mm	53.5	0.1	0.004	0.432	0.52
R-2 mm	21.57	0.44	0.012	0.103	0.11
R-5 mm	63.02	0.44	0.012	0.258	0.26
R-10 mm	100	0.44	0.012	0.516	0.53

## Supplementary Materials

The following are available online at https://www.mdpi.com/1424-8220/20/11/3030/s1, Figures S1–S3 included in the supplementary materials file.
