# Supplementary material for: Continuous-Flow Separation of Magnetic Particles from Biofluids: How Does the Microdevice Geometry Determine the Separation Performance?
Source: Sensors (Basel). 2020 May 27;20(11):3030. doi: 10.3390/s20113030 (PMC7308945; doi:10.3390/s20113030)
Supplement: Supplementary file 1 [file sensors-20-03030-s001.pdf]

Supplementary materials

Continuous-Flow Separation of Magnetic Particles from Biofluids: How Does the Microdevice Geometry Determine the Separation Performance?

Cristina González Fernández [1], Jenifer Gómez Pastora [2], Arantza Basauri [1], Marcos Fallanza [1], Eugenio Bringas [1], Jeffrey J. Chalmers [2] and Inmaculada Ortiz [1,*]

[1] Department of Chemical and Biomolecular Engineering, ETSIIT, University of Cantabria, Avda. Los Castros s/n, 39005 Santander, Spain; gonzalezferc@unican.es (C.G.F.); arantza.basauri@unican.es (A.B.); marcos.fallanza@unican.es (M.F.); eugenio.bringas@unican.es (E.B.)

[2] William G. Lowrie Department of Chemical and Biomolecular Engineering, The Ohio State University, 151 W. Woodruff Ave., Columbus, OH 43210, USA; gomezpastora.1@osu.edu (J.G.P.); chalmers.1@osu.edu (J.J.C.)

* Correspondence: ortizi@unican.es; Tel.: +34-94-220-1585

Received: 16 April 2020; Accepted: 25 May 2020; Published: date

Table of contents

Figure S1. Effect of fluid velocity on bead recovery when the same magnetic force ($F_{m,z}$ = 0.011 nN) is applied for rectangular and U-shaped channels.

Figure S2. Variation of the residence time with the fluid flow rate for all of the studied geometries.

Figure S3. Dependence of bead recovery with the channel length and fluid flow rate for (a) rectangular and (b) U-shaped cross section microdevices.

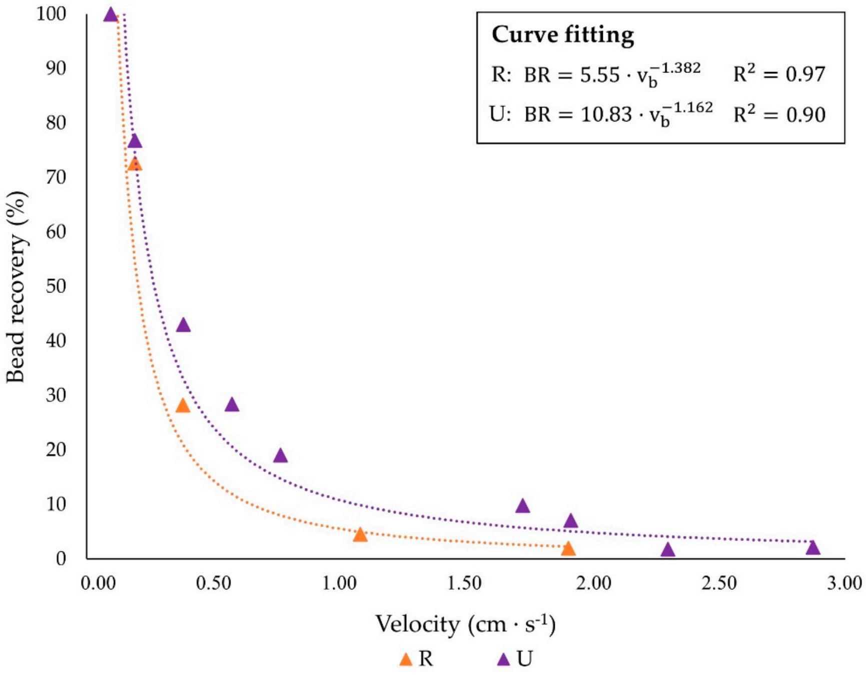

Figure S1. Effect of fluid velocity on bead recovery when the same magnetic force ($F_{m,z}$ = 0.011 nN) is applied for rectangular and U-shaped channels.

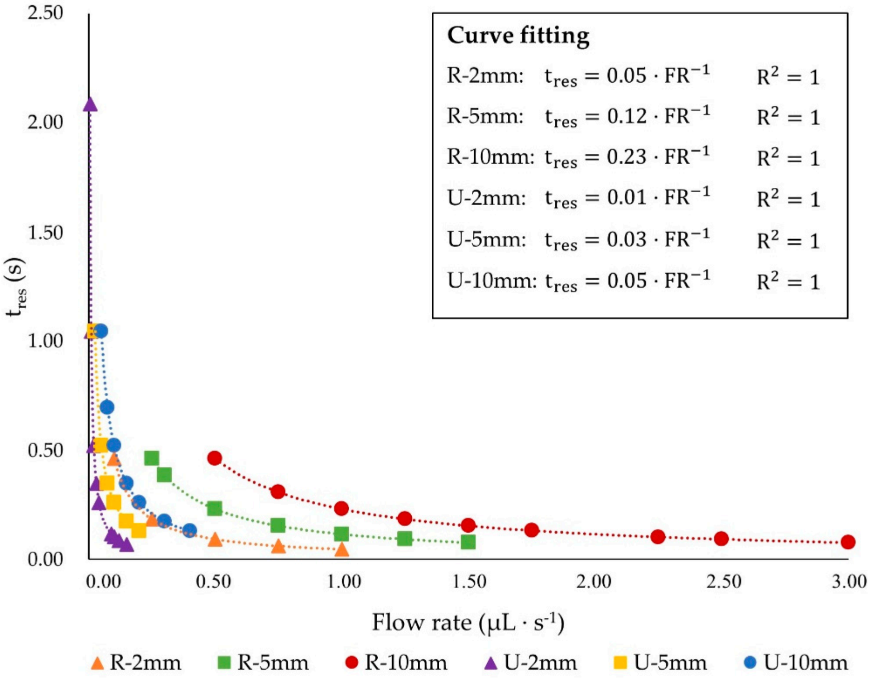

Figure S2. Variation of the residence time with the fluid flow rate for all of the studied geometries.

a)

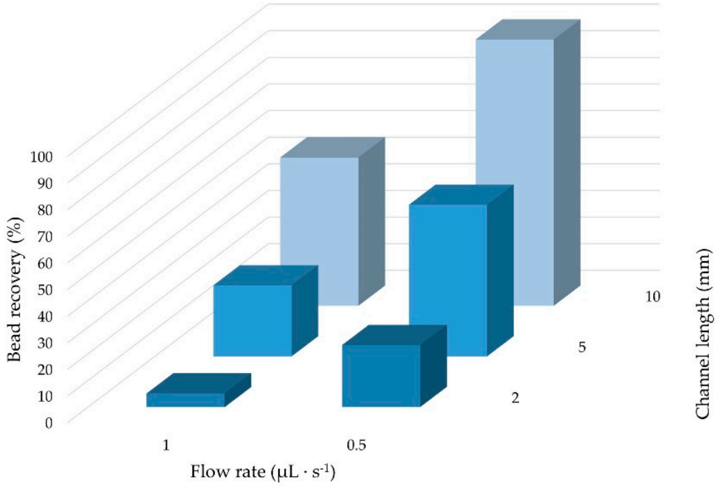

b)

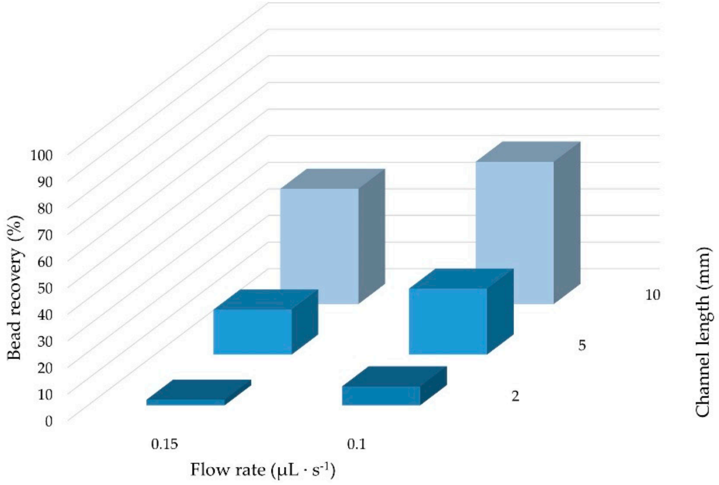

Figure S3. Dependence of bead recovery with the channel length and fluid flow rate for (**a**) rectangular and (**b**) U-shaped cross section microdevices.
